# EDITORIAL

**DOI:** 10.2174/1875397300801010001

**Published:** 2008-02-25

**Authors:** Wei Zheng

We are pleased to launch this inaugural issue of *Current Chemical Genomics*. As an open access online journal, all the papers from this journal can be read free of charge by our readers without any restrictions to access of the full content. This peer-reviewed journal publishes research letters/articles, reviews, and technology notes on all aspects of the research and development focusing on integrative approaches at the interface of chemistry and genomics. The journal reports the newly identified small molecule probes and their applications in chemical genomic research. It also reports on the methods and technologies used to identify and optimize chemical probes that interact with proteins of unknown function and with proteins of cellular signaling pathways. Coverage also includes reports on the new chemo- informatics methods for chemical genomics. We hope that these approaches will facilitate the identification of new drug targets and the development of novel therapeutic strategies leading to a new generation of medicines.

In the last decade, a number of academic screening centers have been established aimed in the discovery of small molecule probes for academic research. The small molecule probes can serve as an alternative tool to unlock the functions of unknown genes and play an important role in genome research. In 2005, the National Institutes of Health (NIH) of the United States launched a probe screening and development initiative named the Molecular Libraries Screening Center Network (MLSCN). These NIH-funded centers carry out high-throughput screenings to identify compounds active in target- and phenotype-based assays; optimize the identified hits into chemical probes, and deposit screening data to a freely accessible public database, PubChem. NIH also established an Imaging Probe Development Center (IPDC) to discover and produce new imaging probes as well as to improve probe detection sensitivity for biomedical researches and clinical applications. Therefore, through all of these efforts, chemical genomics research has moved to a new level. More probes will become available and easier to access for all researchers in the near future. We will publish the selected probes identified from these centers and others including both small molecule and imaging probes.

Open access to all scientific literature is an irresistible trend for the future of scientific publishing. A comparison between the open access and non-open access research papers revealed that the open access publications are more quickly recognized and cited than these non-open ones (Eysenbach G, Plos Biol. 2006,4(5): e157; MacCallum and Parthasarathy, Plos Biol. 2006,4(5): e176). Free online access of scientific articles enables researchers to quickly and effectively search and obtain scientific information and thus efficiently facilitate their research work. To meet the challenge of the electronic era, Bentham Scientific Publishers has decided to launch a series of open access journals including *Current Chemical Genomics*.

With this open access format, we believe that *Current Chemical Genomics *will become an important resource for the information of new probes, methods and technologies on chemical genomics research as well as serve as an alternative journal for quickly publishing research related to chemical genomics. We welcome and encourage our readers to submit suggestions and feedbacks to support us on fulfilling the missions of this new journal.

